# A Missense Mutation (c.1037 G > C, p. R346P) in *PAPSS2* Gene Results in Autosomal Recessive form of Brachyolmia Type 1 (Hobaek Form) in A Consanguineous Family

**DOI:** 10.3390/genes13112096

**Published:** 2022-11-11

**Authors:** Saima Mustafa, Malik Fiaz Hussain, Muhammad Latif, Maryam Ijaz, Muhammad Asif, Mubashir Hassan, Muhammad Faisal, Furhan Iqbal

**Affiliations:** 1 Institute of Pure and Applied Biology, Zoology Division, Bahauddin Zakariya University, Multan 60800, Pakistan; 2 Department of Zoology, Division of Science and Technology, University of Education, Lahore 54770, Pakistan; 3 The Steve and Cindy Rasmussen Institute for Genomic Medicine, Nationwide Children’s Hospital, Columbus, OH 43205, USA; 4 Faculty of Health Studies, University of Bradford, Bradford BD7 1DP, UK

**Keywords:** brachyolmia, WES, sanger sequencing, Western blot, 3D protein structure

## Abstract

Background: Brachyolmia is a skeletal disorder with an autosomal mode of inheritance (both dominant and recessive) in which the patients have a short height, scoliosis and a reduced trunk size. Methods: From the Muzaffargarh District in Pakistan, a consanguineous family with multiple Brachyolmia-affected subjects were enrolled in the present study. Basic epidemiological data and radiographs were collected for the subjects. Whole exome sequencing (WES) which was followed by Sanger sequencing was applied to report the geneticbasic of Brachyolmia. Results: The WES identified a missense mutation (c.1037 G > C, p. R346P) in exon 9 of the *PAPSS2* gene that was confirmed by the Sanger sequencing in the enrolled subjects. The mutation followed a Mendalian pattern with an autosomal recessive inheritance mode. Multiple sequence alignment by Clustal Omega indicated that the *PAPSS2* mutation-containing domain is highly conserved. The HEK293T whole-cell extract that was transfected with the Myc-tagged PCMV6-*PAPSS2* of both the wild and mutant constructs were resolved by SDS-PAGE as well as by a Western blot, which confirmed that there are different PAPSS2 protein expression patterns when they were compared between the control and Brachyolmia patients. This difference between the normal and mutated protein was not evident when the three-dimensional computational structures were generated using homology modeling. Conclusion: We report a missense mutation (c.1037 G > C, p. R346P) in the *PAPSS2* gene that caused Brachyolmia in a consanguineous Pakistani family.

## 1. Introduction

Brachyolmia is a skeletal dysplasia in which the subjects have a short stature and a short trunk with an affected spine [1,2]). Brachyolmia has been characterized into four clinically well-defined types. Brachyolmia type 1 is a name that is given to the Hobaek and Toledo forms. It has an autosomal recessive mode of inheritance. Patients suffering from this type of disease have platyspondyly with a widening of the vertebral bodies, their inter vertebral spaces are irregular and narrow, and they have scoliosis [1,3]. Brachyolmia type 2, also known as Maroteaux type, is similar to type one as it has also an autosomal recessive mode of inheritance, but the patients have rounded vertebral bodies and pedicles that are less over-faced which makes this type different from type 1 [4]. Brachyolmia type 3 transmits in an autosomal dominant form, and the patients have severe kyphoscoliosis, and their cervical vertebrae are irregular and flattened [5]. Brachyolmia type 4 is also known as the spondyloepimetaphyseal dysplasia [SEMD] Pakistani type, and it is an autosomal recessive disorder. The patients have a short stature that is evident at birth, and their lower limbs are usually short and bowed. They are reported to be suffering from mild brachydactyly, enlarged knee joints kyphoscoliosis and precocious osteoarthropathy [2]. 

*PAPSS2* (MIM #603005) is positioned at 10q23.2-q23.31. This gene has 13 exons and generates human 3′-phosphoadenosine 5′-phosphosulfate synthase 2 (PAPS2 protein) [6]. PAPS is involved in the sulfation of a number of molecules in a cell including various steroids and protein hormones as well as glycosaminoglycans (GAGs), and hence, it is considered as a universal donor of sulfate [7]. The sulfation of GAGs plays an important role in the development of the skeletal system [8]. Previously, it has been documented that mutations in *PAPSS2* lead to a functional loss in the PAPSS2 proteins which leads to multiple types of skeletal abnormalities in patients suffering from Brachyolmia [9].

The genetic basis of a short stature with or without skeletal abnormalities has been reported before from Pakistan [10,11,12], but to the best of our knowledge, the genetic basis of Brachyolmia is reported here for the first time from Pakistan. We have applied whole exome sequencing and the Sanger sequencing approach to report the genetic basis of Brachyolmia in an enrolled Pakistani consanguineous family. We are reporting that the Brachyolmia in this family is caused due to a missense mutation in the *PAPSS2* gene.

## 2. Material and Method

### 2.1. Recruitment of Family and Collection of Blood Samples

**Figure 1:**
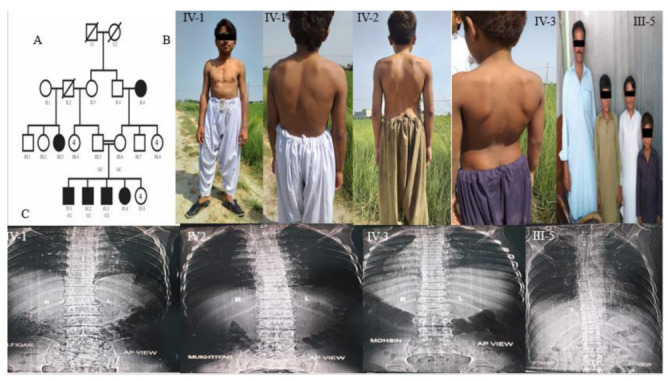
Pedigree and clinical manifestations. (**A**) Pedigrees of a consanguineous Pakistani family segregating autosomal recessive form of brachyolmia. Double lines are indicative of consanguineous union. Clear symbols represent unaffected individuals, while filled symbols represent affected individuals. The diagonal line through a symbol is indicative of a deceased family member. (**B**) Affected individual IV.1 showing disproportionate short trunk and relatively long upper extremities. Individual IV.2 and IV.3 showing mild scoliosis. Individuals IV-1, IV-2 and IV-3 with their unaffected father III-5. (**C**) Radiographic features of brachyolmia. in three patients and one control. Radiograph of spines of affected members IV.1, IV.2 and IV.3 showing mild scoliosis and platyspondyly and irregular end plates. Radiograph of III-5 showing normal spine.

We have enrolled a consanguineous Pakistani family from the Muzaffargarh District in the Punjab province where multiple members have a short stature with severe skeletal malformations. Before the data and blood sample collection, we obtained written informed consents from all of the participants. A questionnaire was filled on sampling site to collected epidemiological data by interviewing each subject. The blood samples were collected from two unaffected and three affected individuals from this family (Figure 1A). The subjects were radiographed (for the thoracic and spinal regions and for the appendicular skeleton) at a commercial laboratory. The genomic DNA was extracted by using a commercial kit (Qiagen, Hilden, Germany) following the instructions that were provided by the manufacturer which were included with the kit.

### 2.2. Whole Exome Sequencing

Two controls (III-5 and III-6) and one patient (IV-2) were selected for the WES from the enrolled family (Figure 1A). The WES and the resultant data from it were analyzed as reported elsewhere [13]. ANNOVAR was used for the variant annotation. The candidate variants were selected as they fulfilled the following criteria: (a) the variant should be nonsynonymous or they have splice sites within 6 base pairs of an exon, (b) the variant should have a minor allele frequency (MAF) that is less than 1% in Kaviar, gnomAD and in-house database, and (c) the variant should co-segregated with the phenotype of the enrolled family.

### 2.3. PCR

In order to confirm the variant that was identified by the WES, the following primer pair: GGATTTGGGTCTTAATGCTT as the forward primer and GGTCCCATTTAGCTGAGGATG was used to amplify exon 9 and its flanking intronic sequence in *PAPSS2* by a PCR from the genomic DNA. The PCR reaction mixture was prepared in a total volume of 50 µL that contained 0.4 µM dNTPs, 2X PCR buffer, 0.3 µM µL of each primer, 5 µL of DNA template with 40 ng/µL concentration and 1 µL of KOD FX taq polymerase (Toyobo, Osaka, Japan). The thermal profile conditions were: an initial denaturation for 2 min at 94 °C, which was followed by 35 cycles of denaturation for 10 s at 98 °C, annealing for 30 s at 55 °C and extension for 45 s at 68 °C. A final extension was carried out for 1 min at 68 °C. The PCR products were resolved during the electrophoresis on 3% Agarose gel.

### 2.4. Sanger Sequencing

The Sanger sequencing was performed by First base (Malaysia): a commercial service provider.

### 2.5. Cloning of PAPSS2 Gene

The forward (GGGCGATCGCATGTCGGGGATCAAGAAGCA, *SfaA1* restriction site underlined) and reverse primers (GGGCGGCCGCGTTCTTCTCCAGGGACCTGT, *NotI* restriction site underlined) were designed by Snapgene and were used to amplify the full length of the human *PAPSS2.* The amplified PCR products were cleaned by using purification kit (Axygene, Guangzhou, China) and then, they were digested with *SfaAI* and *NotI*. The same enzymes were used to digest the PCMV6-Myc-DDK vector. DNA ligase (Thermofisher, Waltham, USA) was used to ligate the digested PCR products and vector to create wild type full-length *PAPSS2.* Finally, the ligated product was transformed into competent *Escherichia coli* (*E. coli*) DH5α cells (Invitrogen, Carlsbad, CA, USA). Colonies that were grown on ampicillin LB agar were selected and they were sequenced to confirm the full-length *PAPSS2* ligation with the expression vector. The PCR-based mutagenesis was used to generate the missense mutation in *PAPSS2* by applying a site-directed mutagenesis method to the wild-type *PAPSS2* expression construct (Heckman and Pease, 2007). Specific primers were designed to check the inserted mutation in the PCMV6-Myc-DDK wild-type full-length *PAPSS2*. The PCR products were purified following their amplification, and then, they were digested with *DpnI,* and the digested products were transformed into competent *E.coli* DH5α cells (Invitrogen, Carlsbad, CA, USA). To confirm the mutation, the colonies that showed growth on the ampicillin LB agar were chosen and sequenced.

### 2.6. Cell Culture, Transfection and Immunoblotting

Dulbecco’s modified Eagle’s medium (DMEM) (Thermo fisher scientific, USA) supplemented with 10% fetal bovine serum and 5% CO_2_ was used to culture the HEK293A cells at 37 °C. The HEK293 cells were plated at a density of 1 × 10^5^ cells/12-well plate and cultured for a day until they reached confluence. Polyethylenimine (PEI) (Thermofisher scientific, Waltham, USA) was used for the transfection, and the cells were used 48 h after the transfection. SDS-PAGE was used to analyze the recombinant proteins from the transfected cells, which was followed by immuno-blotting.

The proteins were separated by SDS-polyacrylamide gel electrophoresis and transferred to a PVDF membrane. The membrane was incubated overnight with a mouse monoclonal antibody against Myc-tag (1:1000; Cell Signaling Technology), which was the primary antibody. After washing it with the TBST buffer, the membrane was incubated for one hour with a secondary antibody, which was mouse monoclonal HRP (1:1000; Cell Signaling Technology). The membrane was further incubated with a chemiluminescent substrate, and finally, it was exposed to a high-performance chemiluminescence film. α view, Protein simple (USA) was used to analyze the film.

### 2.7. Multiple Sequence Alignment Analysis

The PAPSS2 protein sequences from various species were download from an ensemble (https://asia.ensembl.org/index.html, accessed on 26 August 2020). Clustal Omega was used for the analysis of these downloaded multiple sequences.

### 2.8. Protein Model Building

Three-dimensional protein structures of PAPSS2 were computationally designed by fetching their amino acids sequences from Uniprot Knowledge Database (IDs: O95340), and the homology modelling approach was developed to predict the three-dimensional structures of the wild and mutant PAPSS2 protein structures. The automated Swiss modelling approach (https://swissmodel.expasy.org/, accessed on 11 November 2020) was employed to predict the PAPSS2 protein. Two templates (1X6V and 1JDN) with a sequence identity (78.20 and 31.04%) were selected to construct the models.

## 3. Results

### 3.1. Phenotype and Radiographic Findings

Upon the clinical examination and radiography, the affected subjects of the family presented features of Brachyolmia with an autosomal recessive form. The affected subjects had a short stature with a reduced trunk size that became conspicuous during childhood. Their upper extremities were relatively long, and their facial appearances were normal (Figure 1B). The radiographs of the affected individuals displayed rectangular vertebral bodies and mild scoliosis (Figure 1C).

### 3.2. PAPSS2 Gene Mutation

**Figure 2:**
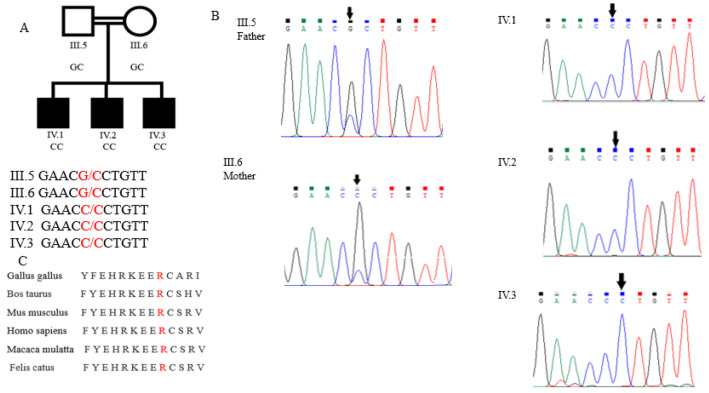
Chromatogram and Clustal Omega alignment of PAPSS2. (**A**) Three individuals (III-5, III-6 and IV-2) were selected for whole exome sequencing. According to the mode of inheritance, individuals with normal height (III-5 and IIIV.6) carried heterozygous alleles (G/C), while affected individuals (IV-2) carried homozygous mutant alleles (C/C). Mutation in five family members was confirmed by Sanger sequencing. (**B**) Chromatogram for PAPSS2-selected region showing c.1037 G  >  C transition. (**C**) Multiple sequence alignment of PAPSS2 from six different organisms performed with Clustal Omega showing R346P (shown in bold) conservation in diverse vertebral species.

The data analysis identified approximately 4880 candidate variants in each trio that were sent for WES. All of the suspected variants were confirmed by the Sanger sequencing. A single candidate gene, *PAPSS2*, was identified with a recessive model of inheritance. A homozygous G-to-C change at the nucleotide position 1037 (c.1037 G  >  C) in exon 9 of *PAPSS2* was detected in the affected individuals of this family. This mutation caused an Arginine-to-Proline amino acid change at the position 346 (p. R346P). The unaffected siblings were heterozygous for this mutation (Figure 2B).

The Clustal Omega analysis revealed that Arginine and the other amino acids of this PAPSS2 protein domain are highly conserved among the vertebrates (Figure 2C), and any mutation in this region may lead to profound effects on the phenotype of the subjects.

### 3.3. Full-Length and Mutated PAPSS2 Gene Expression

**Figure 3:**
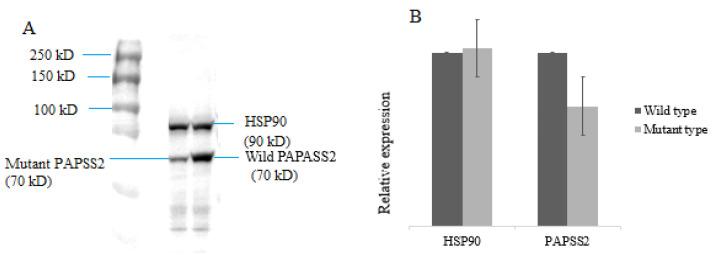
Western blotting and relative Protein expression. (**A**) The 293T cells were transiently transfected with expression plasmids for the PAPSS2 target protein with a DDK-Myc tag. Cells were collected 48 h later, and equal amounts of the whole-cell lysates were subjected to immunoprecipitation with antibodies against the target protein. Lane 2 for mutant type PAPSS2 protein, lane 3 for wild target protein. Lane 1 protein molecular marker of 250 kD. (**B**). Relative expression of proteins. Mutated PAPSS2 protein showing higher expression at 70 kD when it was compared to wild PAPSS2. Housekeeping gene HSP90 showing similar expression in both plasmids (wild type and mutant).

A significantly reduced expression of the mutant protein was observed during the Western blot analysis when it was compared to normal PAPSS2 protein, having approximately a 70 kDa mass (Figure 3A,B).

### 3.4. PAPSS2 Protein Structure Analysis

**Figure 4:**
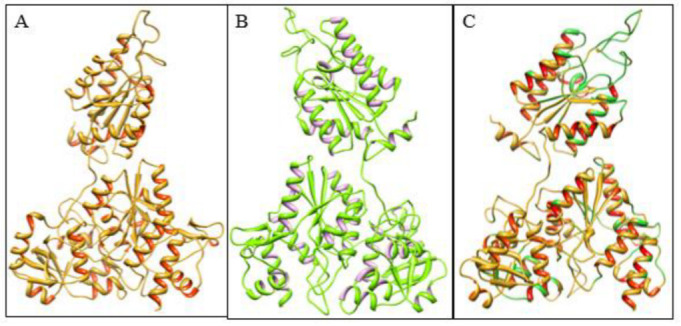
Predicted protein structures for PAPSS2. (**A**) Predicted protein structure for wild PAPSS2. (**B**) Predicted protein structure for mutant PAPSS2. (**C**) Superimposition of wild (orange and red) and mutant (green) structures (PAPSS2).

The superimposition results showed that all of the PAPSS2 protein helices were perfectly aligned in both the wild and mutant structures, and the mutation detected during present investigation did not result in any structural variation in the PAPSS2 protein (Figure 4)。

## 4. Discussion

During the present investigation, we have used WES to identify and report a homozygous mutation in *PAPSS2* that caused an autosomal recessive form of Brachyolmia in an enrolled consanguineous Pakistani family with affected subjects exhibiting a reduced height and skeletal abnormalities. The diagnosis of Brachyolmia in this family was confirmed by a physical examination by a medical team at District Headquarter Hospital Muzaffargarh (Punjab, Pakistan), and the radiographic findings in the affected individuals were complementary to the subjects that were previously reported to be suffering from Brachyolmia from various parts of the world [2,14].

The PAPSS2 protein encodes 3′ phosphoadenosine 5′ phophosulphate synthetase 2 that converts ATP and inorganic sulphate into PAPS (3′ phosphoadenosine 5′ phosphosulphate) [15]. The 2000PAPSS2 protein is important in skeletal development as it appears in chondrocytes during their proliferation and differentiation. In a mouse embryo, PAPSS2 plays its major role between 13.5 and 16.5 days post-conception and beyond this time point, its expression is strongly down regulated in the hypertrophic chondrocytes of mouse embryos [16]. Recently, Brylski et al. [17] have reported that *PAPSS2* disease-related mutations cause the misfolding and aggregation and inhibition of the catalytic function of the protein. It has been suggested that the rare diseases that are associated with *PAPSS2* mutations may, thus, be treated by the supplementation of the compounds that they lack or by using the inhibitors that can reduce aggregation. There are already few studies that are available in literature regarding patients from different ethnic backgrounds that have established thatmutations in the *PAPSS2* gene can result in the autosomal form of Brachyolmia [2,14]. A major contribution on this topic was made by Bownass et al. [18], as they had sequenced *PAPSS2* in 18 patients with different ethnic backgrounds and with their age ranging from infancy to 19 years, and they reported mutations that lead to the autosomal recessive form of Brachyolmia. Among the patients, eight presented prenatally with a short femor, whereas later in childhood, their short spine phenotype emerged, while the others presented with a disproportionately short stature with a short spine that was associated with variable symptoms of pain, stiffness and spinal deformity. Whole exome sequencing was followed by Sanger sequencing which confirmed that most of the enrolled subjects were homozygous for c.809G > A in *PAPSS2* [18]. Although, the enrolled subjects during the present study who are suffering from Brachyolmia are carrying a different mutations (c.1037 G > C, p. R346P) than just the mutation that was reported by Bownass et al. [18], but they share a similar phenotype (the subjects had short stature with a reduced trunk size with relative long upper extremities) as the mutations were harboring the same gene: *PAPSS2.*

Recently, Melissa Perez-Garcia et al. [19] has reported that a novel variant [*p.His496Pro* (*H496P*)] in *PAPSS2* manifests with mild brachyolmia but a disproportionate short stature in male and female Jordanian siblings. The spinal x-rays revealed platyspondyly with disproportionate body measurements. A skeletal survey revealed platyspondyly, with an increasing suspicion of a growth plate pathology. The biochemical phenotype with low circulating levels of dehydroepiandrosterone (DHEA) sulfate and high DHEA levels reflect a sulfonation defect.

In addition to Brachyolmia, earlier mutations of *PAPSS2* have also been reported in patients suffering from spondyloepimetaphyseal dysplasia (SEMD) Pakistani type, which was later on reclassified as Brachyolmia type 4. Tuysuz et al. [20] reported five patients from a Turkish family that were suffering from the SEMD Pakistani type. The enrolled subjects were found to be homozygous for a nonsense mutation (p. R329X) that lead to a premature stop codon formation in *PAPSS2*. This mutation resulted in a precocious costal calcification, a development in the small iliac bones, short femoral necks, coxa vara and fused vertebral bodies in the affected subjects.

Mutations in *PAPSS2* have also been documented in a patient with spondylodysplasia and premature pubarche [21]. A Turkish girl with a short stature, hyperandrogenic anovulation and skeletal dysplasia was compound heterozygous for a missense (143 C > G transversion leading to T48R substitution at a conserved residue in the adenosine 5-prime-phosphosulfate kinase domain) and a nonsense mutation (985C > T transition resulting in R329X) in *PAPSS2*. This quick review of the literature of patients with different ethnic backgrounds confirms that the mutation in *PAPSS2* can lead to an autosomal recessive form of Brachyolmia.

## 5. Conclusions

In conclusion, we have report a missense mutation (c.1037 G  >  C, p. R346P) in exon 9 of *PAPSS2* which caused an autosomal recessive form of Brachyolmia in an enrolled consanguineous Pakistani family. 

## Data Availability

The raw data associated with this project is available at https://www.ncbi.nlm.nih.gov/sra/PRJNA876018, accessed on 7 October 2022.
